# 660. Early Experience with Use of Pemivibart for the Pre-exposure Prophylaxis of Coronavirus Disease 2019

**DOI:** 10.1093/ofid/ofaf695.215

**Published:** 2026-01-11

**Authors:** Brian S Metzger, Jorge R Bernett, Kimberly A Couch, Lucinda J Van Anglen

**Affiliations:** Austin Infectious Disease Consultants, Austin, Texas; Infectious Disease Doctors Medical Group, APC, Walnut Creek, California; Healix, Stevensville, MD; Healix Infusion Therapy, LLC, Sugar Land, Texas

## Abstract

**Background:**

Pemivibart is an investigational monoclonal antibody that is available for emergency use by the U.S. FDA since March 2024 under an emergency use authorization (EUA) for the pre-exposure prophylaxis of COVID-19 in adults and adolescents. Pemivibart is administered every 3 months to patients who have moderate-to-severe immune compromise due to certain medical conditions or receipt of certain immunosuppressive medications or treatments and who are unlikely to mount an adequate immune response to COVID-19 vaccination. The purpose of this study is to report early use of pemivibart.

**Table 1.**
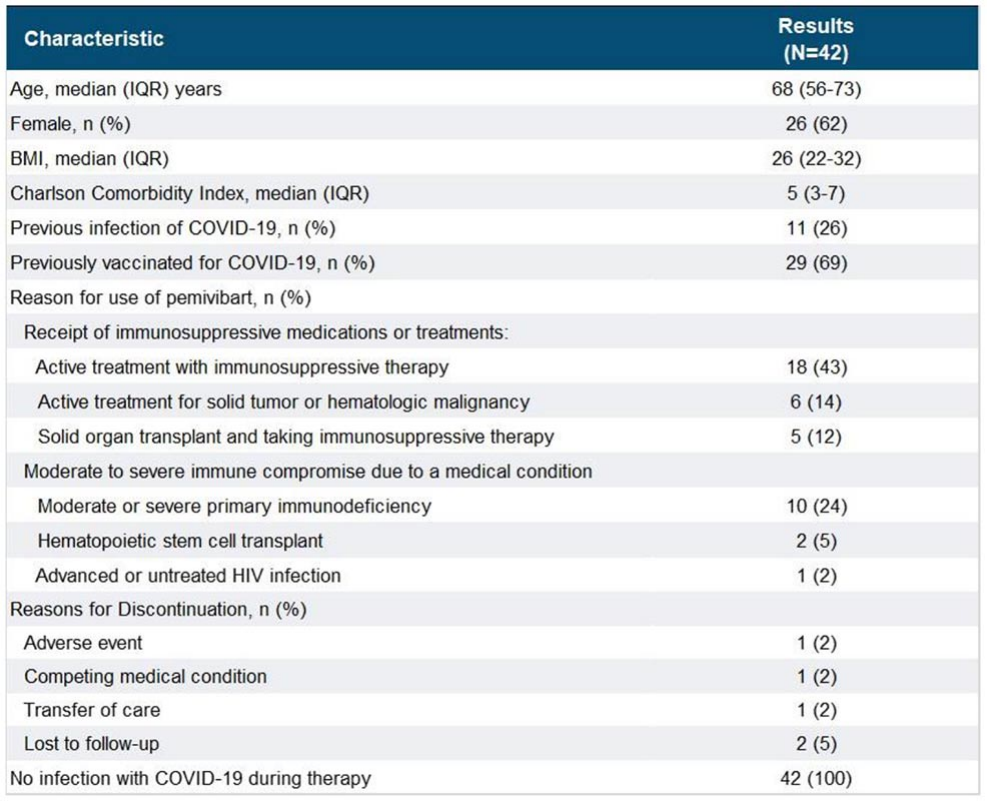
Demographics and Treatment Characteristics

**Methods:**

A retrospective, cohort study was conducted in Infectious Disease and ambulatory infusion centers in patients who received ≥ 1 dose of pemivibart for the pre-exposure prophylaxis of COVID-19. Data collected included demographics, medical history, immune compromised reason for use, treatment details, and adverse events.

**Results:**

Overall, 42 pts received pemivibart following EUA approval from May 2024 through April 2025. Patient and treatment characteristics are shown in Table 1.Median age was 68 years (IQR 56-73). The most prevalent reason for use of pemivibart was active treatment with immunosuppressive medications (n=29, 69%), followed by moderate or severe immune compromise (n=13, 31%). A total of 29 (69%) patients were previously vaccinated for COVID-19, and 11 (26%) had previous episodes of COVID-19 infection. The pemivibart dose was 4500mg in all infusions with 97 infusions administered to date. Pre-medications were administered in 50 (52%) infusions, primarily acetaminophen and diphenhydramine. The majority (97%) of patients were observed for 2 hours following infusions. Of the 42 patients, 37 remain on therapy. Two patients (5%) experienced adverse events, including one with tachycardia and shortness of breath and one with headache. Reasons for discontinuation are noted in Table 1. No COVID-19 infections have been reported in the patients on pemivibart therapy.

**Conclusion:**

Pemivibart has been safely administered in a varied immunocompromised population. Although this is a small cohort, COVID-19 infections have not occurred since initiation of pemivibart. Additional study will be warranted to confirm the continued effectiveness of pemivibart in prevention of COVID-19.

**Disclosures:**

Kimberly A. Couch, PharmD, MA, FIDSA, FASHP, Healix: wages

